# Draft Genome Sequences of Helcococcus ovis Strains Isolated at Time of Metritis Diagnosis from the Uterus of Holstein Dairy Cows

**DOI:** 10.1128/MRA.00402-19

**Published:** 2019-05-30

**Authors:** Federico Cunha, Soo Jin Jeon, Peter Kutzer, KwangCheol Casey Jeong, Klibs N. Galvão

**Affiliations:** a Department of Large Animal Clinical Sciences, College of Veterinary Medicine, University of Florida, Gainesville, Florida, USA; b Landeslabor Berlin-Brandenburg, Frankfurt (Oder), Germany; c Department of Animal Sciences, University of Florida, Gainesville, Florida, USA; d Emerging Pathogens Institute, University of Florida, Gainesville, Florida, USA; e D. H. Barron Reproductive and Perinatal Biology Research Program, University of Florida, Gainesville, Florida, USA; University of Arizona

## Abstract

Helcococcus ovis is an emerging pathogen implicated in the pathogenesis of metritis in dairy cows. Herein, we report the first draft genome sequences of four Helcococcus ovis isolates from the uterus of dairy cows with metritis. This information will enable a better understanding of the bacterium’s pathogenicity and antimicrobial resistance.

## Abstract (precis)

Helcococcus ovis is an emerging pathogen implicated in the pathogenesis of metritis in dairy cows. Herein, we report the first draft genome sequences of four Helcococcus ovis isolates from the uterus of dairy cows with metritis.

## ANNOUNCEMENT

Helcococcus ovis is a fastidious catalase-negative Gram-positive facultatively anaerobic coccus from the family *Peptostreptococcaceae* (1). It has been isolated from bovine and equine pulmonary abscesses, ovine bronchopneumonia and pleuritis, and bovine valvular endocarditis (2–4). Recent studies observed that cows with metritis had a higher abundance of *H. ovis* than healthy cows (5, 6). Despite abundant evidence of the organism’s clinical significance as an animal pathogen, information on the virulence mechanisms of *H. ovis* remains scarce. Here, we performed whole-genome sequencing of four *H. ovis* strains isolated from the uterus of two dairy cows (identification [ID] 8090 and 8749) at the time of their metritis diagnosis.

Sampling was performed in June 2016 in the University of Florida’s Dairy Research Unit in Hague, FL. The swabs were resuspended in 1 ml of Luria-Bertani broth (Sigma-Aldrich), and 200 μl of diluted sample was incubated for 72 hours at 36°C and 6% CO_2_ on *Helcococcus* elective agar (7). Colonies of less than 1 mm in diameter and without pigmentation were individually subcultured on Columbia blood agar with 0.002% pyridoxal HCl for 72 hours at 36°C and 6% CO_2_. Four agar plates with pure cultures were submitted to Genewiz (South Plainfield, NJ) for Sanger sequencing of the 16S rRNA gene. The four isolates were confirmed as *H. ovis* using 16S rRNA gene sequence BLAST comparisons against the NCBI 16S database producing matches with an identity of ≥99% (8). The strains were named KG36 (ID 8749), KG37 (ID 8090), KG39 (ID 8090), and KG40 (ID 8749) and were selected for whole-genome sequencing.

The four isolates confirmed as *H. ovis* were individually cultivated on Columbia blood agar with 0.002% pyridoxal HCl for 72 hours at 36°C and 6% CO_2_, and colonies were scraped for genomic DNA extraction. Genomic DNA extraction was carried out using a DNeasy blood and tissue kit (Qiagen) according to the manufacturer’s instructions. A Nextera XT kit (Illumina, Inc.) was used to prepare the library according to the manufacturer’s instructions. The library was loaded into a MiSeq reagent kit version 2, and sequencing was performed using a MiSeq instrument (Illumina, Inc.) with a 2 × 250-bp 500-cycle cartridge. There were 721,170 reads in KG36 with a genome coverage of 90×, 963,746 reads in KG37 with a genome coverage of 120×, 652,860 reads in KG39 with a genome coverage of 81×, and 1,151,043 reads in KG40 with a genome coverage of 143×. After FastQ data were trimmed using Sickle version 1.33.1 (9) with the length parameter set to 50 and quality set to 30, the reads were *de novo* assembled with SPAdes version 3.11.1 (10) using the k-mer values of 21, 33, 55, 77, 99, and 127. Assembled genomes were annotated using the Prokaryotic Genome Annotation Pipeline (PGAP) (11) and PATRIC (12). Default parameters were used for all software unless otherwise specified. Public GenBank files were annotated with PGAP (11).

Genome statistics and genomic features of the four isolates are listed in Table 1. All the strains contained a ribosomal protection gene (*tetB*) and major facilitator superfamily (MFS) efflux gene (*tetA*), which confer resistance to tetracyclines (13). In particular, KG40 contained AcrEF-TolC, an inner membrane proton/drug antiporter characteristic of Gram-negative bacteria which can confer resistance to fluoroquinolones, cephalosporins, cephamycins, and penams (14). This report will allow for future comparative genome analysis among *H. ovis* strains that cause disease in different body sites or in other species.

**TABLE 1:** Genome statistics and genomic features of *H. ovis* isolates

Isolate	Length (bp)	No. of contigs	*N*_50_ (bp)	GC content (%)	No. of CDSs^a^	No. of rRNAs	No. of tRNAs
KG36	1,770,716	86	128,315	27.56	1,713	4	33
KG37	1,745,991	121	51,850	27.49	1,697	2	33
KG39	1,817,018	131	44,512	27.57	1,771	6	33
KG40	1,778,488	140	43,431	27.67	1,745	5	33

a CDSs, coding DNA sequences.

### Data availability.

The whole-genome sequences are available at DDBJ/EMBL/GenBank under the accession numbers SCFS00000000 (KG36), SCFR00000000 (KG37), SCFQ00000000 (KG39), and SCFP00000000 (KG40). This project and the trimmed reads have been uploaded into the NCBI Sequence Read Archive and can be found under BioProject number PRJNA514352 and SRA numbers SRX5460743 (KG36), SRX5460744 (KG37), SRX5460741 (KG39), and SRX5460742 (KG40).
